# A Novel NOX Inhibitor Alleviates Parkinson’s Disease Pathology in PFF-Injected Mice

**DOI:** 10.3390/ijms241814278

**Published:** 2023-09-19

**Authors:** Kwadwo Ofori, Anurupa Ghosh, Dinesh Kumar Verma, Darice Wheeler, Gabriela Cabrera, Jong-Bok Seo, Yong-Hwan Kim

**Affiliations:** 1 Department of Biological Sciences/Neuroscience Program, Delaware State University, Dover, DE 19901, USA; kofori19@students.desu.edu (K.O.); aghosh@desu.edu (A.G.); dverma@desu.edu (D.K.V.); dbwheeler21@students.desu.edu (D.W.); gncabrera19@students.desu.edu (G.C.); 2 Seoul Center, Korea Basic Science Institute, Seongbuk-gu, Seoul 02841, Republic of Korea; sjb@kbsi.re.kr

**Keywords:** α-synuclein preformed fibrils (PFF), cytotoxicity, reactive oxygen species (ROS), oxidative stress, protein aggregation

## Abstract

Oxidative stress-mediated damage is often a downstream result of Parkinson’s disease (PD), which is marked by sharp decline in dopaminergic neurons within the nigrostriatal regions of the brain, accounting for the symptomatic motor deficits in patients. Regulating the level of oxidative stress may present a beneficial approach in preventing PD pathology. Here, we assessed the efficacy of a nicotinamide adenine phosphate (NADPH) oxidase (NOX) inhibitor, an exogenous reactive oxygen species (ROS) regulator synthesized by Aptabio therapeutics with the specificity to NOX-1, 2 and 4. Utilizing N27 rat dopaminergic cells and C57Bl/6 mice, we confirmed that the exposures of alpha-synuclein preformed fibrils (PFF) induced protein aggregation, a hallmark in PD pathology. In vitro assessment of the novel compound revealed an increase in cell viability and decreases in cytotoxicity, ROS, and protein aggregation (Thioflavin-T stain) against PFF exposure at the optimal concentration of 10 nM. Concomitantly, the oral treatment alleviated motor-deficits in behavioral tests, such as hindlimb clasping, rotarod, pole, nesting and grooming test, via reducing protein aggregation, based on rescued dopaminergic neuronal loss. The suppression of NOX-1, 2 and 4 within the striatum and ventral midbrain regions including Substantia Nigra compacta (SNc) contributed to neuroprotective/recovery effects, making it a potential therapeutic option for PD.

## 1. Introduction

As an idiopathic neurodegenerative disorder, Parkinson’s disease (PD) is characterized by the loss of dopaminergic neurons in the striatum (Str) and Substantia Nigra pars compacta (SNpc), resulting in substantial reduction of dopamine [1]. Although the upstream mechanism of neuronal death is derived from protein aggregates called Lewy body, oxidative stress has been commonly found to mediate the break-down and neuronal loss in the SN in the midbrain in PD pathology [2]. There are numerous medicines available in the market including Levodopa (L-DOPA), which is a common treatment option for PD patients, targeting potential increment and/or normalization of dopamine levels without preventing or reversing neuronal loss. Therefore, there is a high demand for therapeutics, beyond symptom alleviation, needed for retrograding PD progression. Hence, regardless of the upstream causes in PD pathology, targeting oxidative stress pathways may present viable means to reduce cellular oxidative stress in preventing neurodegeneration. A few critical contributors to generate endogenous Reactive Oxygen Species (ROS) are nicotinamide adenine dinucleotide phosphate (NADPH) oxidase (NOX), lipoxygenase, Cyclooxygenase (COX), xanthine oxidase, cytochrome P450 and nitric oxide synthetase [3]. In addition, mitochondrial dysfunctions have been implicated in the pathogenesis of PD and other neurodegenerative diseases, due to the lack of its functional energy generation for cells, especially neurons which require high energy expenditure [4]. Among others, mitochondria dysfunctions are also known to be implicated in impaired calcium buffering, dyshomeostasis, oxidative stress, ROS generation, programmed cell death and other cellular dysfunctions [5]. Here, we focus on targeting NOX inhibition since NOX-mediated ROS generation has been implicated in inducing major tissue damage, resulting from the activation of NOX coupled with increased oxidative stress [6]. The risk of generating excessive ROS can be particularly high during the production of dopamine in the midbrain. During this period, the levels of glutathione (GSH) and intracellular calcium within the SNpc in PD pathology are lower than healthy conditions [7,8]. In this study, we explored the efficacy of a novel NOX inhibitor as a potential therapeutic option for PD treatment. We assessed the inhibition of specific NADPH oxidases to reduce ROS generation for rescuing dopaminergic neuronal loss against the preformed fibrils of alpha-synuclein (PFF) in PD pathology models [9].

The NOX family is made up of transmembrane proteins, known to catalyze the production of superoxide free radicals by transferring an electron to oxygen from electron donating cytosolic NADPH by the action of flavin adenine dinucleotide (FAD) [10]. It has been reported that 7 different isoforms including NOX-1, -2 and -4 are expressed in neurons, while the expression of NOX-2 is known to be high in non-neuronal cells during brain injuries [11,12,13,14,15,16]. Thus, we assessed a novel NOX-1, 2 & 4 inhibitor, the compound-11 (C-11) which is a pyrazole derivative small molecule, as a potential therapeutic to reduce protein aggregation, cellular oxidative stress and a pathological marker, phosphorylated alpha-synuclein (p-α-syn) in nigrostriatal neurons as well as alleviating PFF-induced nigrostriatal neuronal loss in PFF-injected mice. Since our recent publication validated that the inhibition of NOX would be a feasible option as a potential therapeutic for PD [17], our goal is to identify a safer and more effective compound than the previously tested compound (C-6) for a clinical application (See Figure S1). Notably, the NOX inhibition can be an effective approach, not only for inducing neuroprotection in Parkinson’s disease pathology, but also for ameliorating motor deficits in mice.

## 2. Results

### 2.1. A Novel NOX Inhibitor Treatment Decreases Cytotoxicity and Increases Cell Viability in PFF Treated N27P Dopaminergic Cells

**Figure 1:**
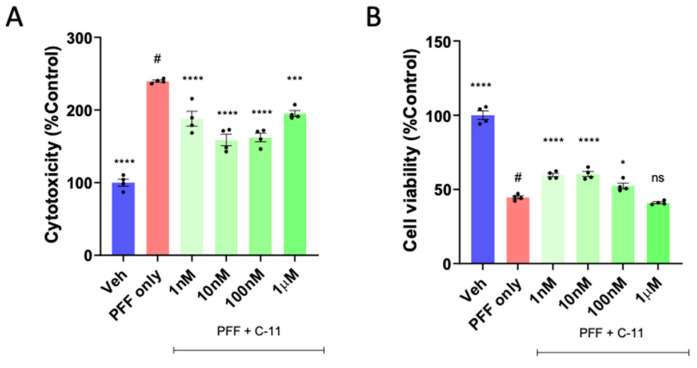
The compound-11 treatment decreases cytotoxicity and increases cell viability in PFF exposed N27P dopaminergic cells. We tested at various concentrations (1 nM–1 µM), with 10 nM as the optimal concentration in the LDH (**A**) and MTT (**B**) assay. One-way ANOVA, Dunnett’s test was applied to compare with PFF only treated cells (#) (n = 4). *: *p* < 0.05, ***: *p* < 0.001 and ****: *p* < 0.0001. ns: not significant.

As we reported earlier [16,17], we found that the preformed fibrils of α-synuclein (PFF) treatment at 1 μg/mL concentration for 24 h induces the generation of reactive oxygen species in N27 dopaminergic cells [16,17,18,19]. We introduced the cells with PFF and then treated with the compound-11 at varying log scale concentrations ranging from 1 nM to 1μM. The compound treatment at various concentrations reduced PFF-induced cytotoxicity in Lactate dehydrogenase (LDH) analyses (Figure 1A) and increased the cell viability in 3-(4,5-dimethylthiazol-2-yl)-2,5-diphenyl-2H-tetrazolium bromide (MTT) analyses (Figure 1B). The results of these analyses reveal that the compound reduced cytotoxicity and improved cell viability at the optimum concentration of 10 nM, which is more effective at lower doses than the previously reported compound (Figure S1) [17].

### 2.2. The C-11 Treatment Decreases Protein Aggregation and ROS Generation Induced by PFF

**Figure 2:**
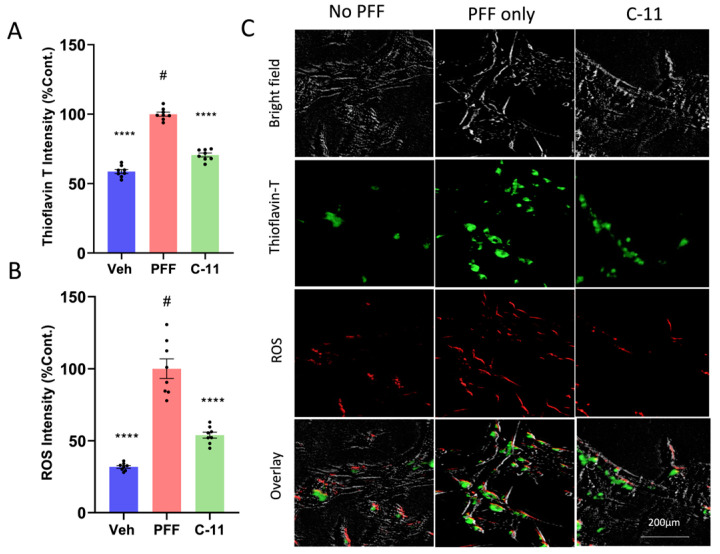
The levels of protein aggregation and reactive oxygen species (ROS) generation induced by PFF are significantly reduced by the C-11 treatment in N27p dopaminergic cells. (**A**) The levels of protein aggregation in Thioflavin T stain in the C-11 treated group was significantly decreased relative to the PFF only control. (**B**) The level of ROS was also reduced in the C-11 treated group, compared to the control. (**C**) Image panels show that PFF-induced ROS generation and protein aggregation were often overlapped and prevented or reversed by the C-11 in N27p cells. One-way ANOVA, Dunnett’s test was applied to compare with PFF only treated cells (#) (n = 8/group in triplicate). ****: *p* < 0.0001.

Next, we investigated the potential cell protective mechanisms by the novel compound. The ROS analysis was performed to measure the level of oxidative damage by 10 nM PFF [20,21,22]. In quantification of measuring the levels of protein aggregation and ROS using image J [23], more than 35% of PFF-induced Thioflavin-T labels was reduced (Figure 2A) and about 50% of ROS generation was prevented or reversed (Figure 2B) by the C-11 treatment, compared to the PFF-only group. In Figure 2C, representative images are displayed to show the contrast of label intensities in protein aggregation (green) and ROS (red) levels between the compound treated groups (n = 8/group) and PFF only (100% in relativity), indicating that there were substantial reductions in both levels in relative to the negative control (PFF only), due to the preventive or recovery effects.

### 2.3. The C-11 Treatment Prevents PFF-Induced Mitochondrial Dysfunctions in N27 Cells

**Figure 3:**
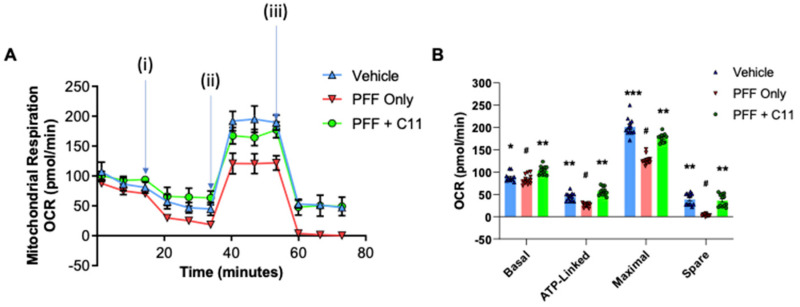
The compound treatment prevents the mitochondrial dysfunction induced by PFF in N27p cells. (**A**) As an indicator of mitochondrial functions, the oxygen consumption rate (OCR) was measured in N27p cells using Seahorse XFe96 extracellular flux analyzer. After an optimal cell density was seeded and stabilized, several reagents are injected as the following order: (**i**) Oligomycin at 1 µM; (**ii**) FCCP at 1µM; (**iii**) Rotenone at 0.5 µM. (**B**) Various mitochondrial respiration functions, such as basal, ATP-linked, maximal with FCCP and spare with rotenone were measured in N27P rat dopaminergic cells. Graphs were exported in prism format from the Seahorse XFe96 analyzer. One-way ANOVA, Dunnett’s test was applied to compare with PFF only treated cells (#) (n = 4/group with 6 wells each). *: *p* < 0.05, **: *p* < 0.01 and ***: *p* < 0.001.

Using the Seahorse XF analyzer (Agilent, Santa Clara, CA, USA), we assessed the potential protective effects on mitochondrial functions in N27 cells by the compound against PFF exposure. The mitochondrial bioenergetic function in N27p cells treated with PFF and the compound-11 was assessed in terms of essential parameters of mitochondrial functions including basal respiration, ATP generation, proton leak, maximal respiration, and spare respiratory capacity, accounting for the levels of oxygen consumption rate (OCR). We exposed the cells to an ATP synthase inhibitor (Oligomycin), an uncoupler (FCCP) and an electron transport chain inhibitor (Rotenone) which is a specific-acting stressor, and these were sequentially injected at specified timepoints. We compared the C-11 treated group with PFF only in addition to the vehicle group. We found that relative to the PFF group, the oxygen consumption rate of the C-11 treated group was significantly higher than that of PFF only group, by which the level was nearly as high as the vehicle group (Figure 3A). In Figure 3B, the effects of the C-11 are demonstrated in comparison with PFF only and vehicle, in terms of the levels of OCR in basal, ATP-linked, maximal with FCCP and spare with rotenone. The OCR analyses were exported from the XFe96 analyzer in GraphPad prism and Excel format.

### 2.4. In Behavioral Analyses, the Oral C-11 Treatment Reduces Motor Deficits in PFF Injected Mice

**Figure 4:**
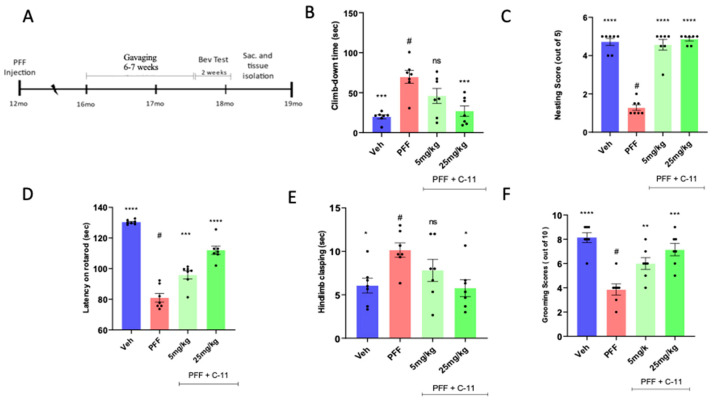
In behavioral analyses, the oral C-11 treatment alleviates motor deficits derived from PFF injection in mice. (**A**) The experimental scheme includes the periods of the compound-11 oral treatment, behavioral tests, and following brain collection. (**B**–**F**) The gavaging for 6–7 weeks improves motor functions in PFF injected mice in behavioral analyses, such as pole (**B**), nesting (**C**), rotarod (**D**), hindlimb clasping (**E**) and grooming (**F**) test. One-way ANOVA, Dunnett’s test was applied to compare with PFF only treated mice (#) (n = 7/group). *: *p* < 0.05, **: *p* < 0.01, ***: *p* < 0.001 and ****: *p* < 0.0001. ns: not significant.

We utilized 12 months aged mice (male and female) purchased from Charles Rivers, since aged mice are more vulnerable to PFF toxicity [16,17,20]. After inoculation, we assessed the mobility of the mice for 4 months to show the potential alleviation from PD-related signs or symptoms in PFF-injected mice. The experimental scheme including gavaging period and behavioral tests is depicted in Figure 4A. In behavioral assessments, we performed several motor coordination tests, such as pole, nesting, rotarod, hindlimb clasping and grooming tests to measure the levels of motility deficits across groups (n = 7/group). Overall, both 5 mg/kg and 25 mg/kg treated mice consistently showed reduced PD-related motor deficits by blinded raters who are not familiar with treatment conditions [24]. For example, in pole tests (Figure 4B), the climb-down time in 25 mg/kg treated mice was significantly shorter than that in PFF only. Likewise, nesting (Figure 4C) and Latency on rotarod (Figure 4D) were substantially improved by both doses. Furthermore, both hindlimb clasping (Figure 4E) and grooming score (Figure 4F) were improved by the treatment in PFF-injected mice [25]. Our results indicate that the compound treated groups exhibited improved motor coordination and skills, as well as motivation, compared to motor deficits displaying PFF only group (#, n = 7/group).

### 2.5. The Neuronal Loss in the STr and SNc Induced by PFF Was Prevented or Partially Reversed by the C-11 Treatment

**Figure 5:**
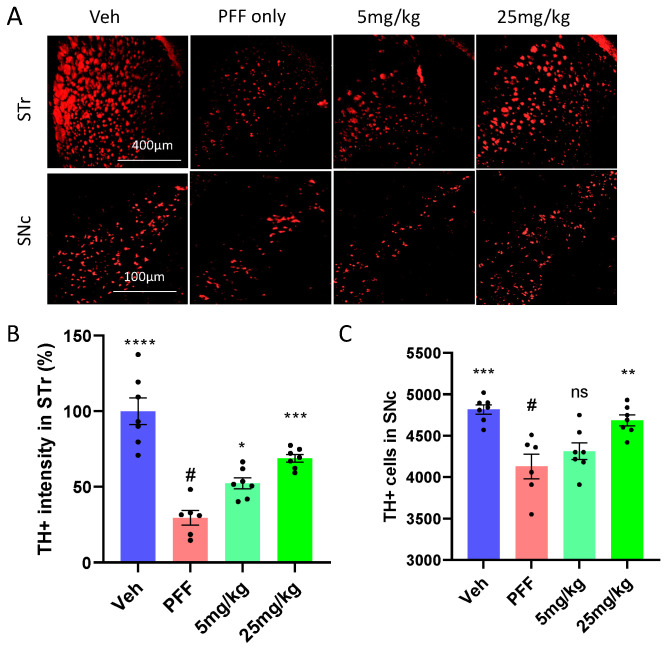
The C-11 treatment results in partial neuronal recovery/protection from PFF induced damage in the STr and SNc. **(A)** The panel of representative images shows immunohistochemical (IHC) images of TH+ stained cells in the striatum (STr) and Substantia Nigra compacta (SNc) regions of mouse brain. The level of TH intensity in the striatum **(B)** and the number of TH+ neurons in the SNc (**C**) show significant neuronal recoveries at both dosage treatments (5 mg/kg and 25 mg/kg), compared to PFF-only group. One-way ANOVA, Dunnett’s test was applied to compare with PFF only treated mice (#) (n = 6–7/group). *: *p* < 0.05, **: *p* < 0.01, ***: *p* < 0.001 and ****: *p* < 0.0001. ns: not significant.

We performed immunohistochemical analyses to determine the levels of protective or recovery effects from PFF inoculation among different groups. First, we stained isolated mouse brain tissues for tyrosine hydroxylase (TH), focusing on the striatum (STr) and substantia nigra compacta (SNc) (Figure 5A). The right hemispheres of the isolated OCT embedded brains were used. Utilizing Image J [23], we quantified the levels of TH-positive cells (TH+) within the STr (Figure 5B) and counted the number of TH+ cells in the SNc in a blind manner (Figure 5C). We found that the C-11 treatment produced some level of protection and partially even reversed the effect of PFF, compared to the neuronal loss induced by PFF in the STr and SNc.

### 2.6. The C-11 Reduces PFF-Induced Protein Aggregates in Thioflavin-T Stain & Phospho-α-syn in the Striatum (STr) and Substantia Nigra Compacta (SNc)

**Figure 6:**
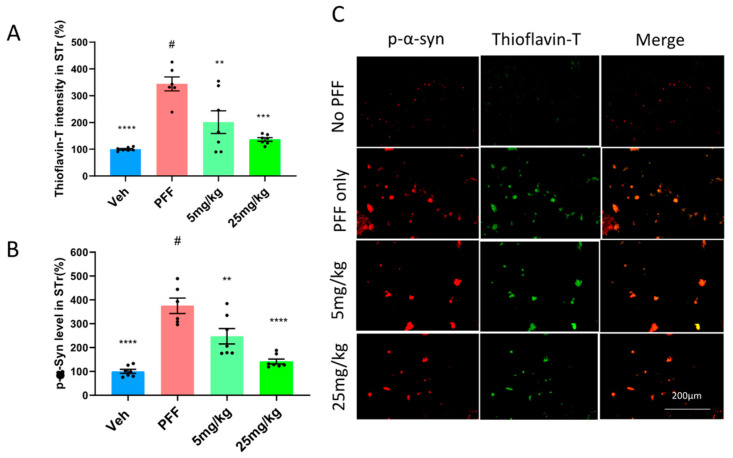
The C-11 reduces PFF-induced protein aggregates in Thioflavin-T stain & phospho-α-syn in the striatum (STr). (**A**) Both doses (5 mg/kg and 25 mg/kg) reduced the level of protein aggregates in Thioflavin-T intensity measurement. (**B**) The oral C-11 treatment significantly decreased the level of phosphorylated-alpha-synuclein in the STr. (**C**) The image panel shows p-α-synuclein (red), Thioflavin-T (green) and merged images as examples. One-way ANOVA, Dunnett’s test was applied to compare with PFF only treated mice (#) (n = 6–7/group). **: *p* < 0.01, ***: *p* < 0.001 and ****: *p* < 0.0001. ns: not significant.

We asked whether the oral compound treatment was effective in decreasing PFF-induced protein aggregation observed in our in vitro assessment (Figure 2A). To investigate this, we performed the colocalization of Thioflavin-T stain and phosphorylated-α-synuclein in IHC. Prior work [22,26] has reported high accumulative levels of p-α-syn in PD patients, accounting for approximately 90% of α-synuclein deposits in Lewy bodies. Here, we assessed the intensity levels of the fluorescent co-labelled Thioflavin-T stain and phosphorylated α-synuclein in the striatum of mouse brains. We found that the C-11 treatment reduced the labeling intensities of Thioflavin-T (Figure 6A) and p-a-syn levels in the striatum (Figure 6B) relative to the PFF group, as shown in Figure 6C for representative images. We confirmed that both 5 mg/kg and 25 mg/kg dosages showed reductions of protein aggregates and phosphorylated Ser-129-α-synuclein (Figure 6A,B). The quantification of label intensities was performed blindly by parties oblivious to experimental parameters or groups.

**Figure 7:**
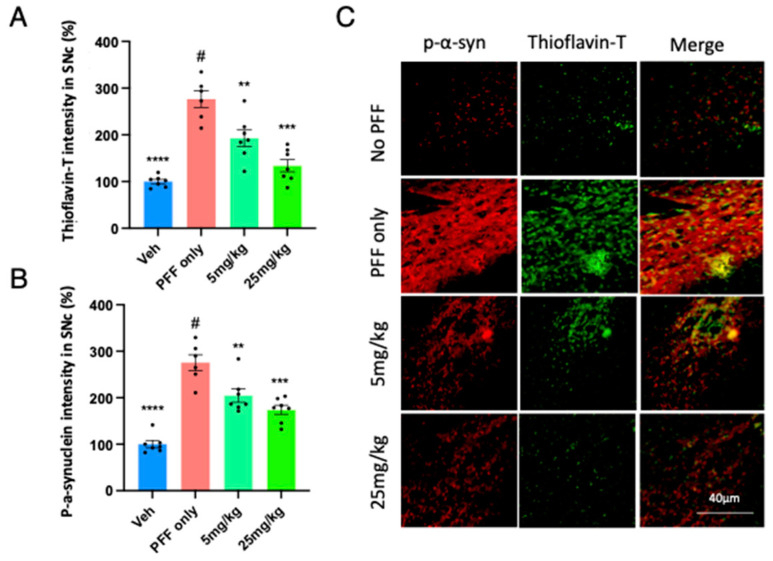
The C-11 treatment reduces the PFF-induced protein aggregation and phosphorylated-Ser129-alpha-synuclein in the SNc. Both doses of the C-11 decreased the label intensity of protein aggregation in Thioflavin-T (**A**) and phosphorylated-alpha-synuclein (**B**). (**C**) The image panel of phospho-α-synuclein (red), Thioflavin-T (green) and merged images in the SNc is displayed as examples. One-way ANOVA, Dunnett’s test was applied to compare with PFF only treated mice (#) (n = 6–7/group). **: *p* < 0.01, ***: *p* < 0.001 and ****: *p* < 0.0001.

Our results from Figure 6 led us to investigate the levels of PFF-induced protein aggregation and phosphorylated-α-syn (p-α-syn) within the substantia nigra. We asked whether we would observe a similar pattern of protein aggregation and p-α-syn within the Substantia Nigra compacta (SNc). We performed the similar co-labelling analyses of Thioflavin-T and p-α-syn to quantify their intensities. We found that the C-11 treatment enabled to mitigate the PFF-induced high levels of protein aggregation (Figure 7A) and phosphorylated-alpha-synuclein (Figure 7B) in the SNc at both 5 mg/kg and 25 mg/kg dosages. In Figure 7C, the image panel of co-labelling of Thioflavin-T and phosphorylated α-synuclein in the SNc of mouse brain is displayed to show heavy co-localization in most cells.

### 2.7. The Levels of Phospho-α-Synuclein, NOX-1, 2, & 4 in the STr and the Ventral Midbrain Were Reduced by the Treatment in Western Blot Analysis

**Figure 8:**
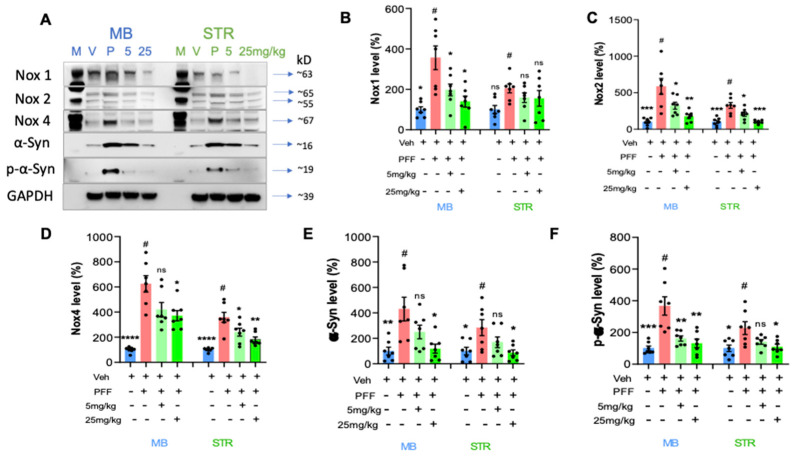
The protein levels of NOX-1, 2, 4, α-syn, and phospho-α-synuclein in the striatum and the ventral midbrain were reduced by the treatment in Western blot analyses. (**A**) Western blot images are depicted to show the levels of proteins of interest and their relative sizes (kD). The band intensity was measured for NOX-1 (**B**), NOX-2 (**C**), NOX-4 (**D**), α-synuclein (**E**) and phospho-α-synuclein (**F**) levels in the midbrain (MB) and striatum (STR). One-way ANOVA, Dunnett’s test was applied to compare with PFF only treated mice (#) (7/group). *: *p* < 0.05, **: *p* < 0.01, ***: *p* < 0.001 and ****: *p* < 0.0001. ns: not significant.

We performed protein level assessment in Western blot analysis using the C-11 treated tissues compared to the PFF only controls [16,17]. We measured the levels of NOX-1, 2, and 4 as well as α-synuclein and phosphorylated Ser-129-α-synuclein in the striatum and ventral midbrain regions of isolated mouse brains as reported in our previous work [17]. As expected with PFF inoculation, the levels of NOX-1, -2, -4, α-syn and p-α-syn were increased in the WB images (Figure 8A). We quantified the band intensities of WB that the C-11 treatment effectively reduced the level of NOX-1 at both treatment dosages within the ventral midbrain (MB), however, there was no statistically significant reduction of NOX-1 in the striatum (STR) (Figure 8B). Next, the level of NOX-2 was significantly decreased by both dosages of the C-11 treatment within the midbrain and striatal regions, relative to the control groups (Figure 8C). We also detected a reduction in the NOX-4 level (Figure 8D), but only at the higher dosage (25 mg/kg) in the midbrain, whereas there were significant reductions in the striatum at both dosages. The C-11 treatments also resulted in reduced levels of both α-syn and phospho-α-syn within the striatal and midbrain regions at 25 mg/kg only, but not 5 mg/kg in Figure 8E,F. However, consistently reduced phospho-α-syn was detected only at the higher dose (25 mg/kg) in both the midbrain and STr. Taken together, our results demonstrate that the compound treatment was efficacious in suppressing the levels of NOX-1, 2 and 4 within the nigrostriatal pathway in the PFF-injected mouse brains.

## 3. Discussion

We previously reported that the preformed fibrils of alpha-synuclein (PFF) induced cellular oxidative stress-related toxicity via the generation of reactive oxygen species (ROS) in N27 rat dopaminergic cells and mouse brains [16,17,21]. Various studies based on pharmacological models of oxidative stress, neuroinflammation and neurodegeneration showed that oxidative and nitrosative stress are critical factors in the pathogenesis and progression of neurodegenerative disorders [27,28,29,30,31,32,33]. In continuation of developing an optimal PD therapeutic in collaboration with AptaBio Therapeutics (Yongin-si, Republic of Korea), we investigated whether another NADPH oxidase (NOX) inhibitor, the C-11 is safer and more efficacious than other NOX inhibitors [17]. We performed the parallel artificial membrane permeability (PAMPA) assay to demonstrate the compound’s permeability in biological membranes (Table S1). The compound is regarded as a pan-NOX inhibitor since the C-11 is an effective inhibitor of NOX-4 as well as NOX-1 & -2, compared to the C-6 [17]. Our in vitro assessments of the compound demonstrated how the compound-11 was effective in reducing the levels of cytotoxicity and reactive oxygen species (ROS) as well as improving cell viability at the optimum concentration of 10 nM (Figure 1 and Figure 2). We also assessed the mitochondrial function of the dopaminergic cells via the utilization of Agilent Seahorse technology to run Mito Stress Test where oxygen consumption rate of cells is measured. A recent publication reported how mitochondrial respiration rate was decreased in PD patient-derived dopaminergic neurons relative to their control group [34]. Another study revealed how mitochondria targeted antioxidant treatment improved mitochondrial function and protected dopaminergic neurons against cell death in a mouse model [35,36]. In this study, we utilized N27P rat dopaminergic cells to assess the mitochondrial functions in vehicle, PFF-treatment, and the compound-11 treated groups. The PFF only group revealed depressed mitochondrial functions, whereas the compound-11 treated group showed significantly recovered mitochondrial functions. Given the integral roles of mitochondria in cellular function and metabolism are critical for homeostasis, the compound may present efficacious means for rescuing from mitochondrial dysfunction caused by PFF treatment, which may be beneficial to maintain homeostasis in cells. Although the C-11 is a potential NOX inhibitor, we are interested in understanding the mechanism of action in mouse brains as well as dopaminergic cells. Our results indicate that the compound suppressed the NOX-1, 2 and 4 protein levels within the ventral midbrain and the striatum, as the various isoforms of NOX have been detected in nigrostriatal regions, when the level of oxidative stress is elevated [10,11,12,13,14,15,37]. The NOX family has also been observed within non-neuronal cells including microglia, as well as in neurons [10,38]. Our interest in this novel NOX inhibitor is due to its universality to target isoforms, capability to cross the blood brain barrier and safety. Previous studies have demonstrated how NOX-2 as a predominant isoform, leads to the downstream expression of NOX-1 and -4 in astrocytes [11,15,39]. Our group reported that the inhibition of NOX-1 and -2 by the treatment of a NOX inhibitor (the C-6) was consistently detected in nigrostriatal neurons, but NOX-4 inhibition was not obviously observed in those regions [16]. Since NOX-4 is one of the seven members of NOX family, it functions as an oxygen sensor and a regulator for cell proliferation, migration and death, in which the increased level is involved in causing cancer metastasis and apoptosis, leading to the development of diverse diseases including hypertension, atherosclerosis, cardiac hypertrophy and other oxidative stress-related illnesses [40]. Here, we report that the inhibition of NOX-1, 2 and 4 by the C-11 may contribute to the efficacy, specificity and safety, and the pan-nature of NOX inhibition by the C-11 would be more effective than the C-6 or other tested NOX inhibitors (Supplementary Figure S1) [17,41]. In addition, the down-stream effect of NOX-1, 2 and 4 inhibitions would be preventing PFF-induced oxidative stress, which is often associated with redox regulation and mitochondrial respiration [8,42]. Therefore, we assessed the potential protective role of NOX inhibition for mitochondrial function including oxygen consumption and found that the treatment prior to the PFF exposure prevented protein aggregation-mediated reduction of mitochondrial respiration [38]. Although the detailed mechanism on how the NOX inhibitor enhances the level of OCR remains to be elucidated, this approach can be effective in reducing or preventing the accumulation of typical PD-related biomarkers, such as protein aggregates, phosphor-α-syn and mitochondrial dysfunctions [26,38,41,43]. Our in vivo data demonstrate that the novel C-11 has the capacity to inhibit NOX-1, 2 and 4 within the ventral midbrain (vMB) at the reported dosages (5 mg/kg and 25 mg/kg). Despite having significant reductive properties in the striatal region for NOX-2 and -4, there was a limited reduction in NOX-1 within the striatum (STr). The anti-oxidative properties of the compound-11 in the down-stream of PD pathology may not only be sufficient to alleviate the PD-related signs, but also play a role in mitigating the progress or even reverse the pathology. Our results suggest that this NOX inhibition not only suppresses the NADPH oxidase enzyme activities, but also reduce the expression in brains, even though we did not quantify the transcriptional levels of those NOX-1, 2 and 4. These in vivo results are promising to encourage us to assess the compound’s effects on other organs including liver and bone, which will be assessed as a follow-up study and to move on next step for the investigational new drug (IND) or clinical trial, however, we reserve more rigorous investigation into its specificity, delivery and mechanism of action for a potential clinical application in future.

## 4. Materials and Methods

### 4.1. Animals

Animal protocols utilized in this study were sanctioned and standardized according to the United States Public Health Service Guide for the Care and Use of Laboratory Animals. All procedures were approved by the Institutional Animal Care and Use Committee (IACUC) at Delaware State University. For in vivo studies, C57BL/6 male and female retired breeder wild-type mice, weighing between (30–50 g), aged at approximately 8–9 months were purchased from Charles River Laboratory (Wilmington, MA). The mice were further housed in a vivarium for an additional 3–4 months in a single animal per polyacrylic cage with access to food and water *ad libitum* and maintained in standard housing conditions, i.e., room temperature 24 ± 1 °C and humidity 60–65% with 12:12, light: dark cycle. At 12 months old, the mice were stereotaxically inoculated with α-synuclein preformed fibrils (PFF). Behavioral assessment was performed 2–3 months after the injection to assess classic parkinsonian symptoms, such as hindlimb clasping, rotarod, etc. An approximation of about 60–70% mice were required to demonstrate PD-related signs to progress unto the compound treatment [16,17].

### 4.2. Cell Culture and Treatment

We utilized N27 parental rat dopaminergic cell line which was obtained from EMD Millipore (Burlington, MA) for in vitro assays/analyses. To assess the effect of the compound treatment, the cell passage number of 11 or below was used. Cells were then treated with 5 μg/mL of wheat germ agglutinin (WGA) (L-9640, Sigma-Aldrich) in media and incubated for 1 h to open cellular pores. The media with WGA was added with 0.1 M N-Acetylglucosamine (PHR-1432, Sigma-Aldrich) at 1 μg/mL and then incubated for 24 h to induce cellular stress for the treatment with 1 μg/mL of pre-formed fibrils of α-syn (PFF) from StressMarq Biosciences (SPR-324C, Victoria, BC, CAN). Per the type of assay, cells were seeded in either 96-well or 6-well plates with the density of 4000 and 200,000 cells, respectively, with a minimum of 4 wells allotted for each treatment (n = 4/group). The compound was dissolved in DMSO and administered to PFF treated cells at varying concentrations to find an optimum concentration for recovery after 24 h of exposure [16,17].

### 4.3. Cell Viability Assay

In a colorimetric assay, 3-(4,5-dimethylthiazol-2-yl)-2,5-Diphenyltetrazolium bromide (MTT) depends on cellular reduction of tetrazolium salts to formazan crystals. For an optimal cell density, we seeded ~4000 cells/well in a 96 well plate for the MTT assay. The MTT solution (ab211091, Abcam) was added and incubated at 37 °C for 3 h for an additional period per the manufacturer’s instructions. Resulting formazan crystals were dissolved in 100% DMSO, leading to a purple color change, and the plate was read at 570 nm wavelength by a spectrophotometer (SpectraMax M5^e^, Molecular devices). We compared the optical density values of the treated cells versus that of the control cells as a percentage of cell viability [17].

### 4.4. Cytotoxicity Assay

Lactate dehydrogenase (LDH) is an enzyme found in almost all cells to be measured for the rate of dead cells. The test or assay measures LDH levels released into media during cellular stress or damage for assessing cytotoxicity using reagents from Pierce LDH cytotoxicity assay kit (88954, Thermo, Waltham, MA, USA). LDH solution was prepared as the manufacturer’s instructions, e.g., 50 μL of media from each well was put into wells of a new 96 well plate and followed by mixing 50 μL of the LDH reagent into each well. The resulting 100 μL of media containing media + LDH reagent was incubated at 37 °C for 30 min, and then applied with stop solution. The mixture was analyzed at 490 nm with the reference wavelength of 680 nm using a spectrophotometer (SpectraMax M5^e^, Molecular devices) [17].

### 4.5. ROS Levels and Thioflavin-T assessment

Cellular ROS and protein aggregation are implicated in PD pathology generated from several sources [18,24,43]. We performed both ROS and Thioflavin-T assay to assess levels of protein aggregation and ROS generation after the exposure of 10 nM PFF. For oxidative stress level detection, cells were incubated in media containing 5 μg/mL of the ROS reagent CellROX deep red reagent (C10422, Thermo Fisher Scientific, Waltham, MA, USA) and 25 μg/mL of Thioflavin-T reagent (CAS:2390-54-7, Acros Organics, Waltham, MA, USA) at 37 °C for 30 min as previously reported [17]. The cells were then washed 3 times with phosphate buffered saline (PBS) without Ca^2+^ or Mg^2+^ (137 mM NaCl, 2.7 mM KCl, 4.3mM Na_2_HPO_4_ and 1.47 mM KH_2_PO_4_). Utilizing the EVOS FL Cell imaging Systems (Invitrogen, Waltham, MA, USA), several images from each well were collected for analyses. With a minimum of 2 wells assigned to each group, and 4–5 times independently repeated experiments (total replicated: n = 8–10/group), the intensity of red fluorescence corresponding to ROS levels and green labels corresponding to protein aggregation were analyzed in a blinded manner using the Image J [23].

### 4.6. Oxygen Consumption Rate (OCR) Measurements Using Agilent Seahorse XF Analyzer

We measured oxygen consumption rate of our N27P cells at 37 °C utilizing a Seahorse XFe96 Extracellular Flux Analyzer (Santa Clara, CA, USA) [42]. Cells were cultured and treated with 10 nM PFF and the C-11 (100 nM) in Agilent Seahorse XeF96/XF Pro cell culture microplates, and then washed once with RPMI assay medium, followed by incubating for 1 h, prior to measurements. The RPMI assay medium consisted of 0.5 mL 1 M glucose solution (Agilent, cat # 103577-100), 0.5 mL 100 mM pyruvate solution (Agilent, cat # 103578- 100), 0.5 mL GlutaMAX-1 (100X, Gibco: 35050-061), and 48.5 mL XF RPMI medium, (pH 7.4, part # 103576-100). A day prior to running the assay, we hydrated sensor cartridge in XF calibrant (part # 100-840-00) and incubated in a non-CO_2_ incubator at 37 °C overnight. Oligomycin, Trifluoromethoxy carbonylcyanide phenylhydrazone (FCCP) and rotenone were prepared for assay from stocks to a final concentration of 1μM, 1μM and 0.5μM, respectively and sequentially injected by the Seahorse XFe96 analyzer after loading them into the sensor cartridge ports per the manufacturer’s directions (Agilent technologies, Santa Clara, CA, USA). The automated system mixed and measured the mitochondrial function of the cells and generated a file to be exported for presentation.

### 4.7. Stereotaxic α-Synuclein PFF Injection and Brain Isolation

All C57BL/6 wild type male and female mice were purchased from the Charles River (Wilmington, MA, USA), at 8–9 month-old and aged until 12 months for stereotaxic injection with 2 μL of mouse α-syn PFF (2.5 μg/μL in PBS, 5 μg each side, SPR-324, StressMarq) in the dorsal striatum bilaterally, as reported previously [17]. The coordinates for injection sites are anterior-posterior (AP) +0.5 mm, medial-lateral (ML) 1.8 mm and dorsal-ventral (DV) 3.5 mm.

### 4.8. Gavaging Schedule

The PFF injected wild type mice were daily administered with the oral treatment at two different doses: 5 mg/kg or 25 mg/kg for 6–7 weeks. Dosage volumes were adjusted each week based on weights of mice as they were routinely monitored upon the compound treatment. Prior and during gavaging, the PFF injected mice were monitored to assess the level of motility deficits and recovery individually. The extended gavaging time was applied to comprehend whether the ongoing neuronal loss because of PFF toxicity can be mitigated with the compound administration, as previously reported [17].

### 4.9. Tissue Isolation

All the mice were anesthetized in 4% isoflurane chamber, then intra-cardially perfused with ice-cold 0.9% saline, followed by 150 mM NaCl/70% Ethanol. We isolated the whole brains from mice carefully after decapitation. The right hemispheres of the brains were kept in 4% paraformaldehyde for 48 h for post-fixing, and then transferred into 30% sucrose solution. We utilized the OCT to embed those half brains, and then sectioned using a cryostat (Thermo) for immunohistochemical analysis. The other hemispheres (left) were region-specifically isolated (e.g., striatum and ventral midbrain) for Western Blot analysis to assess the levels of protein expression [41,42].

### 4.10. Behavioral Assays

#### 4.10.1. Hindlimb Clasping

Healthy wild type adult mice extend all four paws while they are picked up by a tail over a flat surface to ensure a safe position for secure landing [44]. Hindlimb clasping is characterized by motor deficits, which can be evident as “bat-like clasping” stretch of the hindlimbs [17]. In hindlimb clasping assessment, mice are placed on a horizontal plane, held at 2 cm from the tail tip, and then slowly brought up to an elevation of 10 cm from the surface. A 15 s video recording was captured for each mouse for 3 consecutive trial days. In a blinded manner, we then analyzed total time spent in hindlimb clasping for severity.

#### 4.10.2. Rotarod

Rotarod is a behavioral test used to measure motor functionality as an estimate of neuromuscular coordination. Animals were placed on a horizontal rod that rotates about its long axis; the animal is expected to walk forward to remain upright and not falling off [45,46]. After a habituation period of 3 days on a rotating rod with a horizontal plane at a rotating speed of 4 rotations per minute (rpm), the assessment was done with 2 rpm increment every minute for 10 min or until a mouse fell off the rod at 20 rpm, as previously reported [17].

#### 4.10.3. Pole Test

The pole test was used to assess the level of motility deficits. It evaluates mouse ability to grasp and maneuver on a pole to descend into their cages or to the base of the pole. When the pole test was performed, a mouse was placed head-upward on the top of a rough-surfaced vertical pole, and the time until the mouse descends to the floor was recorded to assess the locomotor activity [25]. They were habituated to face down to the pole to climb down for at least 5 days, as reported earlier [17]. On the test day, we noted the time taken for a mouse to make a turn and posit for a descent as well as the latency for the climb-down. A fall off or slip down the pole without turning is considered a failure. For analyses, videos of the test were captured and analyzed in a blinded fashion to get the total climb-down time or the turn time. A minimum of 5 trials were performed for each animal, and then averaged as the final score for data presentation.

#### 4.10.4. Nesting

Nest building has been known to assess the level of neurological dysfunction in PD mouse models. For building a complete nest, mice are required to have orofacial (mouth and face) as well as forelimb movement, which is a determinant of DAergic activity or mechanism [47]. As a nesting tool, a 3 × 3 cm cotton patch was placed in a clean cage per animal. For assessment, we scored the ability of a mouse to tear up and make a nest from the cotton patch. We obtained pictures per cage to assign a score blindly on a scale of 1–5 points where 1 is the least (barely touched/torn up a nesting patch) and 5 (perfectly made nest), the highest score a mouse could attain. The scores were then tallied and represented on a graph to depict nesting capacities after treatments across groups.

#### 4.10.5. Grooming

One of the most common behaviors observed in animals is grooming, which comprises the ability of taking care of the body and fur [48]. It is not considered learned but innate. In general, clean fur is a determinant of good health in mice. After housed individually, the mice were assessed for grooming scores ranging from 1 to 10 with 1 being poor/dull fur coat and 10 being vibrant, shiny and healthy fur coat. The coat conditions of the mice were correlated to levels of motivation and activity of each animal. Dull and non-shiny coats detected represented severe motor deficits, whereas healthy animals showed otherwise as previously reported [17]. To demonstrate grooming uniformity among groups after treatment, mice images were obtained and then for 3 days after treatments. In a blinded fashion, the grooming scores were averaged and analyzed for graphical representation.

### 4.11. Immunohistochemistry (IHC)

Immunohistochemical assay was performed on all experimental groups (Vehicle, PFF, 5 mg/kg and 25 mg/kg) using half mouse brains of right hemispheres in 14μm thickness coronal sections. A 0.1 M PB solution is prepared from 1 M Sodium Phosphate dibasic and 1 M Sodium Phosphate monobasic. Tissue sections were thawed and rehydrated in 0.1 M PB for ~10 min, followed by a permeabilization step (using prechilled 100% acetone, then 40 μL Triton-X in 2% BSA). The sections were then blocked in 2% BSA for 1 h. Primary antibodies from EMD Millipore or Thermo scientific were applied and incubated at 4 °C overnight. Prior to applying secondary antibodies (2′AB) (1:1000; Molecular Probes, Thermo Fisher Scientific), three times washing with 0.1 M PB-T (Triton-X) was performed. A following washing and curing step were performed after secondary Ab incubation with antifade mounting media (Thermo). Glass cover slips were carefully placed on tissue sections, then imaged utilizing EVOS FL Cell imaging Systems (Invitrogen) or confocal microscopy (Zeiss). We measured the intensity levels of tyrosine hydroxylase (TH) (1:500, Cell signaling), phosphorylated Ser-129-α-synuclein (1:500, Cell signaling) and protein aggregation in Thioflavin-T stain (20 μM, Acros) with image J. Images acquired were analyzed in a blinded manner as previously reported [16,17].

### 4.12. Immunoblot Analysis

We performed Western blot (WB) analyses with five proteins of interest: NOX-1, 2, 4, alpha-synuclein and phosphorylated alpha-synuclein. For the immunoblot assessment, we harnessed isolated frozen region-specific tissues from the left hemisphere of mouse brains which were kept at −80 °C. RIPA buffer containing the low concentration of detergent (25 mM Tris-HCL, 150 mM NaCl, 1% NP-40, 1% sodium deoxycholate and 0.1% SDS) was added with protease inhibitors (100 μL for 10 mL) and 5 mM EDTA. We then sonicated the tissue + RIPA mixture for 5 min total time: Pulse ON = 5 sec and Pulse OFF = 10 sec at an amplitude of 25%. After centrifugation at 16,000 rpm, we run a protein estimation assay, BCA analyses to determine protein concentration per sample using a spectrophotometer (SpectraMax M5^e^, Molecular Devices) as described in our earlier work [24]. Samples to run WB were prepared using RIPA and 6x Laemmli dye, and heated at 95 °C for ~5 min. After samples were loaded and electrophoresed, a pre-cast polyacrylamide gel (SurePAGE Bis-Tris, 8%–15%, 12 wells; GenScript, Piscataway, NJ, USA) was transferred to PVDF membrane (Immobilon-P, EMD Millipore, Burlington, MA, USA). Each primary antibody per protein of interest was diluted at 1:1000 (p-Ser-129-α-synuclein and α-synuclein, Cell signaling, cat# 23706S, and 4179S/51510S; NOX-1, 2, 4 rabbit antibodies, Proteintech 17772-1-AP, 14347- 1-AP, and 19013-1-AP) and incubated at 4 °C overnight per a manufacturer’s recommendations. Next, we washed the membranes and treated them with their respective secondary antibodies (1:2000; Invitrogen-31462) at room temperature for 2 hrs. The blots were then developed in immobilon Forte Western HRP substrate (WBLUF0500, EMD Millipore) for the detection under the ChemiDoc iBright CL1500 (Invitrogen). We used GAPDH (1:5000) as a loading control, and PageRuler pre-stained protein ladder on the immunoblots (EMD Millipore-MPSTD4; Thermo Fisher-26616) to verify the molecular weights for each protein, and ImageJ was used to analyze the intensities of specific bands, in comparison with a loading control (GAPDH) for each band to normalize the band intensity, as reported in our previous works [16,17].

### 4.13. Statistical Analyses

In this study, we employed one-way ANOVA Dunnett’s test to assess the effects of the novel C-11 treatment, relative to the PFF-only treated control group (Figure 1, Figure 2, Figure 3, Figure 4, Figure 5, Figure 6, Figure 7 and Figure 8). Most in vitro experiments were run in triple or quadruple with each set including n = 2–3/experiment (n = 8–9/group). In mouse results, n = 6–7/group for behavioral and IHC analyses. When p-values are below 0.05, the results are considered as significant (*). ns: no significance.

## 5. Conclusions

Our in vitro experiments revealed that at the optimal concentration of 10 nM, the C-11 was effective in reducing cytotoxicity, protein aggregation and ROS generation, while it improves cell viability and mitochondrial function, compared to the PFF-only group. In vivo results are well aligned with in vitro findings, demonstrating that the compound treatment rescued PFF-induced behavioral deficits in motility, in addition to decreasing tissue-specific protein aggregation and neuronal loss in brains.

## Data Availability

We make all associated data with this study available and included. **i**n this publication.

## Supplementary Materials

The following supporting information can be downloaded at: https://www.mdpi.com/article/10.3390/ijms241814278/s1.
