# Supplementary material for: A Novel NOX Inhibitor Alleviates Parkinson’s Disease Pathology in PFF-Injected Mice
Source: Int J Mol Sci. 2023 Sep 19;24(18):14278. doi: 10.3390/ijms241814278 (PMC10531511; doi:10.3390/ijms241814278)
Supplement: Supplementary file 1 [file ijms-24-14278-s001.zip › ijms-2587250-supplementary.pdf]

Supplemental Data

Article

A novel NOX inhibitor alleviates Parkinson's disease pathology in PFF-injected mice.

Kwadwo Ofori[1], Anurupa Ghosh[1], Dinesh Kumar Verma[1], Darice Wheeler[1], Gabriela Cabrera[1], Jong-Bok Seo[2] and Yong-Hwan Kim[1]*.

Citation: To be added by editorial staff during production

Academic Editor: Firstname Lastname

Received: date
Revised: date
Accepted: date
Published: date

Copyright: © 2023 by the authors. Submitted for possible open access publication under the terms and conditions of the Creative Commons Attribution (CC BY) license (https://creativecommons.org/licenses/by/4.0/).

Publisher's Note: MDPI stays neutral with regard to jurisdictional claims in published maps and institutional affiliations.

[1]Department of Biological Sciences/Neuroscience program, Delaware State University, Dover, DE 19901, USA.
[2]Seoul Center, Korea Basic Science Institute, Seongbuk-gu, Seoul 02841, Korea.
* Correspondence: yhkim@desu.edu(YHK)*

Table S1.

Group	Conc. (uM)	pH	P_e (10^{-6} cm/sec)
**Progesterone**	50	7.4	35.00±0.34
**Ranitidine**	50	7.4	0
**Compound-11**	12.5	7.4	6.01 ±0.22

P_e (10^{-6}cm/sec)	BBB	P_e (10^{-6}cm/sec)	CNS
< 0.4	Low		
> 0.4	High	>10<br>< 10	CNS+<br>CNS-

We used Parallel artificial membrane permeability (PAMPA) assay as a high-throughput screening technique to predict the permeability of compound 11 relative to progesterone and ranitidine across a biological membrane.

Figure S1.

We performed a cell viability and cytotoxicity comparative assessment of compound-11 and compound-6 to confirm the superior efficacy of compound-11 relative to compound-6 at the optimal concentrations. We found that compound-11 was effective in recovering cell viability at lower concentrations compared compound-6 which was toxic at lower concentrations.

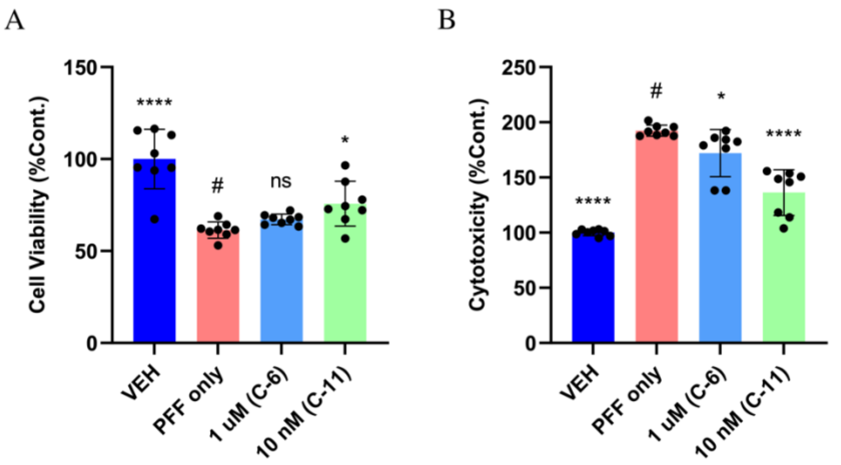

The compound-11 and 6 treatment decreases cytotoxicity and increases cell viability in PFF exposed N27P dopaminergic cells at their optimal concentration. One-way ANOVA, Dunnett's test was applied to compare with PFF only treated cells (#) (n=3/group in quadruple). *: $p<0.05$, ***: $p<0.001$ and ****: $p<0.0001$. ns: not significant.
